# Supplementary material for: A high-resolution mRNA expression time course of embryonic development in zebrafish
Source: eLife. 2017 Nov 16;6:e30860. doi: 10.7554/eLife.30860 (PMC5690287; doi:10.7554/eLife.30860)
Supplement: Supplementary file 6. [file elife-30860-supp6.zip › biolayout-clusters-files/Cluster177-genes.html]

Cluster177


# Cluster177: Genes

| Ensembl ID | Gene Name | Chr | Start | End | Biotype |
| --- | --- | --- | --- | --- | --- |
| ENSDARG00000006915 | arl8ba | 6 | 42954424 | 42973648 | protein\_coding |
| ENSDARG00000015006 | dnm1l | 25 | 20131919 | 20161028 | protein\_coding |
| ENSDARG00000068650 | fam214b | 5 | 18517994 | 18548705 | protein\_coding |
| ENSDARG00000018882 | jak2b | 5 | 65364705 | 65439291 | protein\_coding |
| ENSDARG00000089232 | prr14 | 3 | 32711080 | 32723657 | protein\_coding |
| ENSDARG00000063254 | r3hdm4 | 11 | 5724367 | 5733874 | protein\_coding |
| ENSDARG00000090512 | trim8a | 13 | 11743671 | 11795748 | protein\_coding |
| ENSDARG00000101242 | zcchc14 | 25 | 12483150 | 12538866 | protein\_coding |
